# Pilot Study of an Integrative New Tool for Studying Clinical Outcome Discrimination in Acute Leukemia

**DOI:** 10.3389/fonc.2019.00245

**Published:** 2019-04-09

**Authors:** María José Gacha-Garay, Andrés Felipe Niño-Joya, Natalia I. Bolaños, Lina Abenoza, Guillermo Quintero, Humberto Ibarra, John M. Gonzalez, Verónica Akle, Zayra V. Garavito-Aguilar

**Affiliations:** ^1^Laboratory of Developmental Biology, Department of Biological Sciences, Universidad de los Andes, Bogotá, Colombia; ^2^Biomedical Sciences Group, School of Medicine, Universidad de los Andes, Bogotá, Colombia; ^3^Department of Oncology, Fundación Santa Fe de Bogotá, Bogotá, Colombia; ^4^Microscopy Core, Vice-Presidency of Research, Universidad de los Andes, Bogotá, Colombia; ^5^Laboratory of Neuroscience and Circadian Rhythms, School of Medicine, Universidad de los Andes, Bogotá, Colombia

**Keywords:** zebrafish, acute leukemia, patient-derived xenograft, leukemic stem cell, aldehyde dehydrogenase, translational research, cancer

## Abstract

Acute leukemia is a heterogeneous set of diseases affecting children and adults. Current prognostic factors are not accurate predictors of the clinical outcome of adult patients and the stratification of risk groups remains insufficient. For that reason, this study proposes a multifactorial analysis which integrates clinical parameters, *ex vivo* tumor characterization and behavioral *in vivo* analysis in zebrafish. This model represents a new approach to understand leukemic primary cells behavior and features associated with aggressiveness and metastatic potential. Xenotransplantation of primary samples from patients newly diagnosed with acute leukemia in zebrafish embryos at 48 hpf was used to asses survival rate, dissemination pattern, and metastatic potential. Seven samples from young adults classified in adverse, favorable or intermediate risk group were characterized. Tumor heterogeneity defined by Leukemic stem cell (LSC) proportion, was performed by metabolic and cell membrane biomarkers characterization. Thus, our work combines all these parameters with a robust quantification strategy that provides important information about leukemia biology, their relationship with specific niches and the existent inter and intra-tumor heterogeneity in acute leukemia. In regard to prognostic factors, leukemic stem cell proportion and Patient-derived xenografts (PDX) migration into zebrafish were the variables with highest weights for the prediction analysis. Higher ALDH activity, less differentiated cells and a broader and random migration pattern are related with worse clinical outcome after induction chemotherapy. This model also recapitulates multiple aspects of human acute leukemia and therefore is a promising tool to be employed not only for preclinical studies but also supposes a new tool with a higher resolution compared to traditional methods for an accurate stratification of patients into worse or favorable clinical outcome.

## Introduction

Acute leukemia is a set of heterogeneous diseases characterized by the transformation of hematopoietic stem and precursors cells into malignant cells (1, 2). This results in the recruitment of aberrant leukocyte as blasts, affecting either myeloid or lymphocytic lineages and causing pancytopenia and abnormal high number of blasts in peripheral blood (1, 3).

Acute Myeloid Leukemia (AML) and Acute Lymphoblastic Leukemia (ALL) are considered rare diseases, however, they represent a public health problem due to the increase in their incidence rates over the last decade (1, 4, 5). Additionally, despite the technological advances, current treatments are inefficient, the prognosis of the patients remain poor and the overall five-year survival rate is low between 30–40% for young adults (6, 7).

Acute leukemia comprises a variety of manifestations and is currently classified according to two systems. The French-American-British (FAB) classification based on morphology, lineage and blasts immunophenotype and the World Health Organization (WHO) classification based on cytogenetic and clinical features (1, 6, 8, 9). Both systems are used not only to discriminate between leukemia subtypes but also for the stratification of patients in risks groups (9, 10). However, most of the patients are assigned to intermediate risk, in which the clinical outcome is variable and the prognosis is difficult to predict (11, 12). Due to the intrinsic variability of acute leukemia, its etiology and biology are not completely clear becoming a challenge to understand the behavior of this disease.

During the last decades, new tools have been developed to identify relevant molecular and cellular factors associated with leukemia heterogeneity, initiation, and progression. Many of these studies have characterized LSC as an important leukemogenic factor (13–15). This cell population plays an important role in cell growth maintenance and the reconstitution of the hematopoietic neoplasia particularly after a stressful stimulus such as the induction chemotherapy (16, 17). Multiple biomarkers have been identified and used for the characterization of this cells such as membrane markers (CD34, CD38, CD123, TIM3), transcription factors (NF-κB, HIF-1α), and metabolic markers (enzyme activity, intracellular reactive oxygen species) (15). Recently, evidence has been presented for a clinically relevant marker in the characterization of LSC, the enzyme Aldehyde dehydrogenase (ALDH). This enzyme catalyzes retinaldehyde oxidation to retinoic acid and is mainly expressed in immature cells. Commonly, combinations of cell surface markers and high ALDH activity are used for cancer stem cells identification. Additionally, an increase in ALDH activity has also been related to cancer stem cells and has presented a direct relation to elevated rates of metastasis and tumor growth (18–20).

Although this approach seems to be accurate, in some cases *in vitro* analysis presented significant restraints in their potential to predict and model the biology and therapeutic outcome of cancer (21). For that reason, zebrafish has been proposed as a new model to clarify the mechanisms of initiation, progression, and maintenance of these pathologies. This is due to its multiple biological and experimental advantages for the study of normal or altered hematopoiesis (22–24).

Zebrafish has proven to be an ideal model for testing cancer xenografts not only for the transparency of their embryos that facilitate *in vivo* monitoring but also for the late maturation of the adaptive immune system, their rapid development with relatively short generation time, high fecundity, similar lifespan (2.5 years) compared to mice and lower maintenance costs (25–28). Hematopoiesis and leukemogenesis is also a highly conserved process among vertebrates and the biology of cancer between organisms share cellular and molecular components like cell cycle genes, tumor suppressors and oncogenes (22, 29–32). In addition, zebrafish is a useful tool for the study of biological processes associated to cancer initiation and progression such as senescence and inflammation (33, 34). This animal model has enabled the application of forward genetics to cancer research, and mutations could be easily recapitulated in zebrafish using CRISPR/Cas9 technology or transgenic systems which had helped to identify events involved in carcinogenesis and tumor progression. This has contributed to important insights into cancer pathogenesis and in the development of novel discoveries and approaches to novel therapies (35–37). In addition, these studies have allowed understanding some effects of heterogeneity and the influence of the microenvironment on different types of cancer (24, 38–40).

Considering zebrafish advantages, the importance of LSC and the necessity for more efficient assays that could predict accurately the therapeutic outcome of the patients, in this study, we sought to establish an improved translational model by the integration of basic and patient-oriented research in order to model the behavior of acute leukemia patient-derived xenografts (PDXs) into zebrafish embryos and to establish their relationship with the clinical outcome.

Xenografting tumor cells into animal models are not a new approach; however, their predictive potential regarding clinical outcome remains undefined. This study proposed a pilot study of a new tool for a reliable and accurate stratification of patients with acute leukemia based on an integrative model of leukemia behavior, cell characterization, and clinical features, in addition, to an evaluation of intra-tumor and inter-tumor heterogeneity. Together our approach allows us to introduce an integrative quantitative approach to use zebrafish and tumor characterization as a prediction tool for the behavior of acute leukemia in young adults.

## Materials and Methods

### Animal Care and Handling

Zebrafish wild-type (A/B and TAB5) adults were raised and maintained according to standard conditions with oxygen supply to keep it at 6.0–8.0 ppm (41). Embryos were maintained at 28.5°C in egg water before injection and treated at 6 hpf with 100 μM 1-phenyl 2-thiourea (PTU) for 5 days. At 36 hpf, they were manually dechorionated and treated in PTU. This study was carried out in accordance with the recommendations of the IACUC of Universidad de los Andes. The protocol was approved by the IACUC of Universidad de los Andes (C.FUA_14-010).

### Clinical Information

Seven samples from adult patients newly diagnosed with AL and treatment naïve were collected after patients provided informed consents. Prognostic parameters (age, karyotype, number of blasts, leukocyte, and platelets count), morphological remission status after induction treatment (Table 1), cytogenetic information and induction chemotherapy protocols (Table 2) were obtained from Fundación Santa Fe de Bogotá. Five samples from patients with AML with a mean age of 55 (SE ± 9.6) corresponding to AML subtypes M1, M3, M4, M4Eo, and M5b were used. The remaining two samples were from patients diagnosed with common B-ALL with a mean age of 42(SE ± 8). All the samples were categorized according to the Medical Research Council classification into adverse, favorable or intermediate risk (Table 1). This study was carried out in accordance with the recommendations of Fundación Santa Fe de Bogotá Ethics Committee with written informed consent from all subjects. All subjects gave written informed consent in accordance with the Declaration of Helsinki. The protocol was approved by the Fundación Santa Fe de Bogotá Ethics Committee Act N° CCEI-3888-2015.

**Table 1:** Diagnostic information of patients with acute leukemia.

**Patient**	**Age**	**Subtype**	**% blasts**	**Platelets (K/μl)**	**Leukocytes (K/μl)**	**Cytogenetics**	**Risk stratification**	**Remission**
LPZ6	76–80	M4	16	42	16	Complex	Adverse	No
LPZ10	76–80	M4Eos	42	282	2,2	Normal	Intermediate	NA
LPZ12	40–45	M3	2	35	2	t(15:17)	Favorable	Yes
LPZ13	30–35	M1	84,6	207	90	Normal	Intermediate	No
LPZ15	40–45	M5B	6	29	104,9	47, XY, +6	Intermediate	No
LPZ14	30–35	ALL-B	91,34	26	5,7	Normal	Standard	Yes
LPZ21	46–50	ALL-B	76	231	67,4	47,XXY, t(9;22)(q34;qll)	Adverse	No

**Table 2:** Molecular data and induction treatments of patients diagnosed with acute leukemia at Fundación Santa Fe de Bogota.

**Patient**	**Karyotype**	**Method**	**Molecular evaluation**	**Result**	**Induction therapy**
LPZ6	42,XY,-5,del(7)(q32q34),-8,add(13)(p11.2),-13,add(15)(p11.2),add(16)(q24),add(17)(p13),-18,-19,+mar [18]/46,XY[2]	PCR	t(9;22)(q34;q11) BCR-ABL	–	Not initiated (Dead by pancytopenia complications)
LPZ10	46, XX	PCR	t(9;22)(q34;q11) BCR-ABL	–	NA (Treatment in another institution)
		PCR	t(4;11)(q21;q23), MLL-AF4	–	
LPZ12	46, XY	FISH	t(15;17) (q22;q21) , PML/RARA	+	ATRA^*^ 45 mg/m in 2 divided doses daily + arsenic trioxide 0.15 mg/kg IV (30 days)
LPZ13	46, XY	FISH	t(15;17) (q22;q21) , PML/RARA	–	7 + 3 Protocol: Cytarabine (Ara-C) 200 mg/m^2^ daily continuous infusion × 7 days with idarubicin 12 mg/m^2^ × 3 days.
			t(8;21), RUNX1/RUNX1T1	–	
		PCR/Sequencing	FLT3-ITD	–	
LPZ14	46, XX	FISH	t(4;11)(q21;q23), MLL-AF4	–	GRAALL 2005 Protocol^**^
			t(9;22)(q34;q11) BCR-ABL	–	
			t(1;19)(q23;p13), E2A-PBX1	–	
LPZ15	47, XY, +6	FISH	t(8;21), RUNX1/RUNX1T1	–	7+3 Protocol: Cytarabine (Ara-C) 200 mg/m^2^ daily continuous infusion × 7 days with idarubicin 12 mg/m^2^ × 3 days.
			t(9;22), BCR-ABL	–	
			t(11q23), KMT2A rearrangements	–	
		PCR/Sequencing	Point mutation Exon 15 Codon 645	+	
			FLT3-ITD	–	
LPZ21	47,XXY,t(9;22)(q34;qll)	PCR	t(9;22)(q34;q11) BCR-ABL	+	Dasatinib 140 mg/day + GRAALL 2005 Protocol
		FISH	t(11q23), KMT2A rearrangements	–	
			t(1;19)(q23;p13), E2A-PBX1	–	
			t(12;21), TEL-AML1	–	

* *ATRA, All-trans retinoic acid*.

** *GRAALL 2005 Protocol: Initiation: Vincristine 2 mg/day (day 1,8), prednisone 60 mg/m^2^/day (day 1 to 14), daunorubicin 50 mg/m^2^/day (days 1, 2, 3), L-asparaginase 6,000 UI/m^2^/day (days 8, 10, 12), cyclophosphamide 750 mg/m^2^/day (day 1), intrathecal therapy: methrotrexate (MTX) 15 mg + cytarabine (Ara-C) 40 mg + DEPOMEDROL 40 mg (days 1, 8). Second part: vincristine 2 mg/day (days 15, 22), daunorubicin 30 mg/m*.

### Sample Collection

Primary leukemic samples were obtained from bone marrow aspiration and peripheral blood was collected from healthy donors. Human cell lines erythroleukemia K562 and promonocytic leukemia U937 (provided by Colombian District Hemocenter) were used in this study.

### Cell Isolation and Culture

Primary mononuclear cells were isolated by density-gradient centrifugation (Ficoll-Hypaque). Primary and cell lines were cultured in RPMI 1640 media supplemented with 10% heat-inactivated fetal bovine serum (GIBCO®), CO2 and 37°C.

### Establishment of Leukemic Cells With Stem Cell Properties

LSCs in each sample were labeled with ALDEFLUOR reagent system according to the manufacturer's protocol (Stem Cell Technologies, Vancouver, BC, Canada) and BD anti-CD34 PerCP-Cy5.5 antibodies (Becton Dickinson, San Jose, CA). Flow cytometry assay was performed using a BD FACSCanto II cytometer. The acquisition was performed for 50,000 events approximately and analysis was driven with FACSDiva 6.1.3™ software and FlowJO 10.4.1®. First, we excluded non-viable cells to select the study population in a Forward Scatter—A vs. Side Scatter—A plot. Second, doublets were excluded using a Forward Scatter—H vs. Forward Scatter—A (FSC-H vs. SSC-A) plot and finally, an SSC-A vs. FITC-A plot was used to delimitate *ALDH*^*bright*^*SSC*^*low*^ cells. To conclude, CD34 expression in *ALDH*^*bright*^*SSC*^*low*^ (+) and *ALDH*^*bright*^*SSC*^*low*^ (-) cells were quantified using PerCP-Cy5.5 fluorescence. Diethylamino benzaldehyde (DEAB), a broad inhibitor of ALDH, was used as a control of the background fluorescence.

### Cell Tracking With Carboxyfluorescein Succinimidyl Ester (CFSE)

For cellular tracing, each sample was stained with CFSE (CellTrace™ CFSE Cell Proliferation Kit, Invitrogen, Eugene, USA) as previously reported (42).

### Zebrafish Xenografts Injection

Zebrafish embryos of 48 hpf were anesthetized with 0.04 mg/ml tricaine and disposed on a 10 cm Petri dish covered with 1% agarose. Then 200–500 human cells were injected into the fish pericardial space (PCS). Embryos were maintained at 28.5°C for 1 h. Next, embryos were transferred to 24-well plates and maintained at 32.5°C. Twenty-four embryos with successful injection were used for each sample (non-leukemic, K562, U937, M1, M3, M4, M4Eos, M5, and both B-ALL samples).

### *In vivo* Imaging

Embryos were mounted for live imaging over a cover glass with 1% low melting point agarose and covered with 0.03% tricaine in egg water. Fluorescent image acquisition was performed using an Olympus FV1000 confocal microscope using a 10x/0.4 objective and a filter for Alexa fluor488. Confocal stacks were acquired every 4.2 μm and processed for maximum intensity projections. Images were analyzed with Fiji software (43).

### Human Xenografted Cell Quantification

To determine xenograft efficiency each, of 24 embryos per human sample, with green CFSE fluorescent foci was manually counted for each sample after 1-day post-injection (dpi). On the other hand, the xenograft survival rate was determined by counting each green CFSE fluorescent foci in each of six randomly selected embryos per human sample at 1 and 2 dpi. The spatial dispersion pattern was established for each leukemia subtype. The patterns were compared using a standard deviational ellipse after bootstrapping.

### Xenografted Cells Migration

Foci position was determined based on ten anatomic regions established according to zebrafish hemodynamics (**Figure 4A**). In order to determine if there is a preferential site of migration, the probability to find individual foci in each region was calculated in six randomly selected embryos per human sample.

### Statistical Analysis

Statistical analysis was performed using R software. Statistical differences in survival rates were determined by Bayesian 95% Highest Density Interval. Not-overlap probability distributions represent significant differences between groups. Bootstrapping of foci coordinates and standard deviation ellipse was used to determine statistical differences between spatial dispersion patterns. Not overlapping confidence means significant differences. Cell tropism was analyzed by calculating the probability to find cells in each region. This probability was calculated as the number of events in each region over the total events. Bayesian density distribution was performed for each cell type in order to determine significant differences between anatomical regions. No significant differences between regions corresponded to a random distribution while significant differences corresponded to a clumped distribution. To establish existent correlations logistic regression and multiple logistic regression was performed with a significance value of 0.05. For original data, please contact zv.garavito@uniandes.edu.co.

### Integrative Analytical Tool for Predicting Clinical Outcome

To construct an appropriate and robust predictive model for the clinical outcome of patients with acute leukemia, LSC characterization, PDX behavior and clinical features from patients were analyzed together using a Partial Least Square regression (PLS). This analysis allows combining the relative weight of each variable as a function of the outcome. Thus, PLS prediction is a function of all of the input factors assayed.

## Results

### Cancer Cell Phenotype Ensures Higher Engraftment Rate

Primary samples and cell lines K562 and U937 were injected in the PCS of zebrafish embryos at 48 hpf. Human leukemic and healthy human blood cells were detectable in zebrafish embryos and the success of the engraftment was defined as the percentage embryos with fluorescent foci at 1 dpi. According to our results, xenograft efficiency depended on the type of implanted cells: AML 87.8%(SE ± 7.47), ALL-B 72.5%(SE ± 6,5) and Non-leukemic cells 66%(SE ± 4). Then we determined if the engraftment efficiency has a relation with the clinical outcome of the patients with acute leukemia after induction treatment. Despite samples from patients with a successful morphological remission presented a lower number of engrafted embryos than samples from patients with unsuccessful remission, the differences between both groups are not significantly different (Wilcoxon rank sum test *p*-value = 0.1333; *n* = 24 embryos per sample).

**Figure 1:**
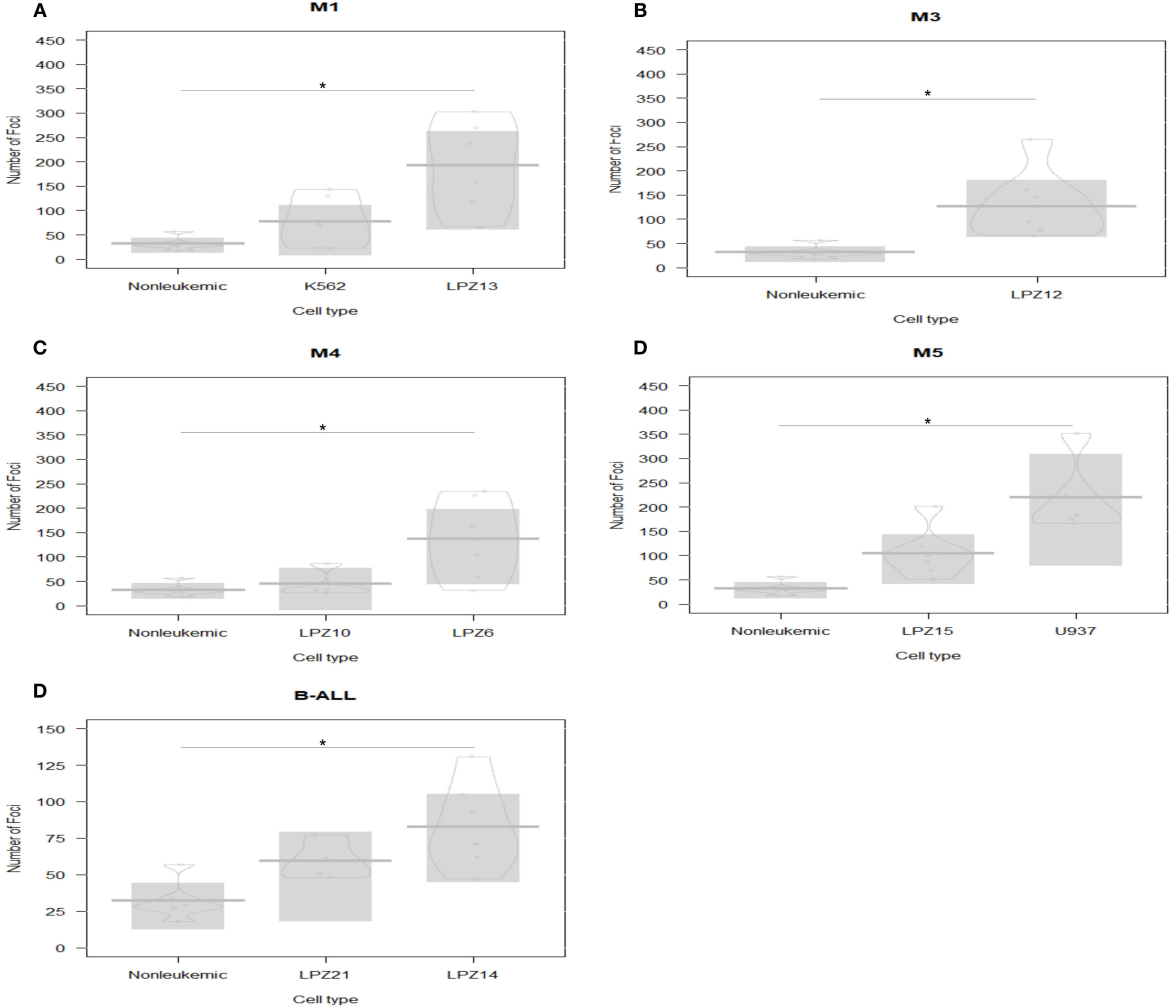
Xenografts survival rate comparison between acute leukemia subtypes based on FAB scheme classification at 1 dpi. PDX and cell line xenografts from leukemic samples presented higher survival rates than non-leukemic xenografts. The highest rates corresponded to the less differentiated leukemia subtypes. **(A)** M1 samples: LPZ13, cell line K562, and non-leukemic cells. **(B)** M3 sample (LPZ12) and non-leukemic cells. **(C)** M4 samples: LPZ6 and LPZ10 compared to non-leukemic cells. **(D)** M5 samples: LPZ15, U935, and non-leukemic cells. **(E)** B-ALL samples: LPZ14 and LPZ21 and non-leukemic cells. The comparison was established according to morphological and immunophenotypic similarities between samples from the same subtype based on FAB scheme classification. Significant differences were determined according to Bayesian 95% Highest Density Interval (*n* = 24 embryos per cell sample). *Non overlapping 95% Bayesian Highest Density Interval.

On the other hand, human-engrafted cell survival rate was defined as the total number of conspicuous foci in each embryo after 1 dpi. First, our results showed a significantly higher survival rate of leukemic cells, compared to healthy blood samples (Figure 1). Additionally, we evidenced that highest survival rates of primary cells corresponded to minimally differentiated subtypes (LPZ13-M1, LPZ12-M3, and LPZ6-M4), suggesting that FAB system has a clinical relevance, lately underestimated (Figures 1A–C). For ALL, leukemic cells presented higher survival rates than controls. When comparing both B-ALL samples, LPZ14 had a higher survival rate, while LPZ21 xenografts were less perceptible; however, this was not statistically significant (Figure 1E).

Second, differences in survival rates between PDX and cell lines were evident (Figures 1A,D). In addition, differences among PDX with similar immunophenotype (FAB system) were observed (Figures 1C,E). In this case, primary cells presented heterogeneous behaviors, mainly associated with the intrinsic variation among patients. This underlines the importance of inter-tumor heterogeneity in this model. However, survival rates were not significantly different.

### Less Differentiated Leukemia Subtypes Have Higher Dissemination Potential

We estimated the migration potential of human cells xenotransplanted into zebrafish embryos after 1 dpi. For that aim, each embryo was analyzed as a coordinate plane in which the site of injection corresponded to the (0, 0) position, the X-axis to the anteroposterior axis, and the Y-axis corresponded to the dorsoventral axis.

**Figure 2:**
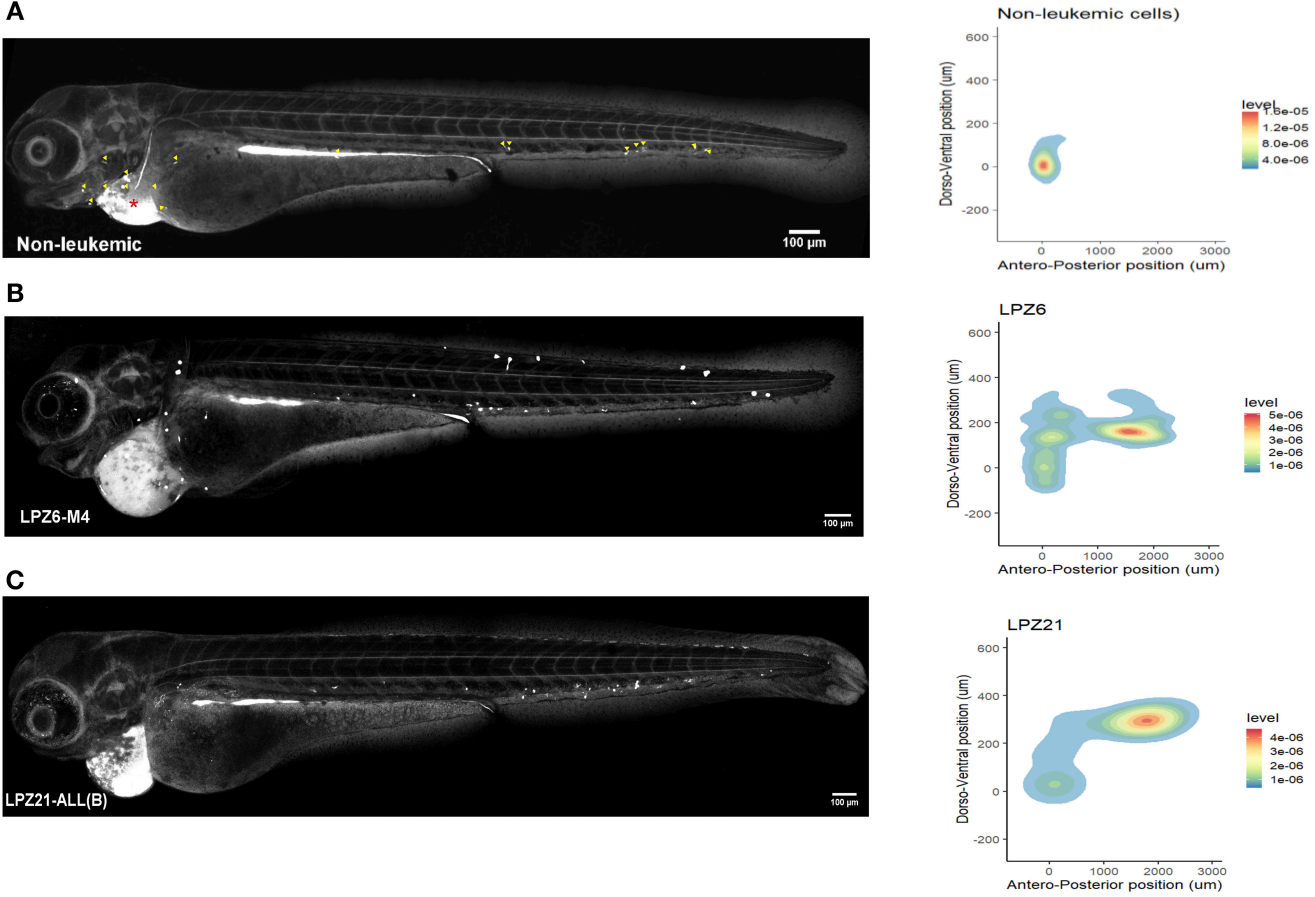
Spatial dispersion pattern of xenografted human cells along zebrafish embryos. The migration of human cells into the zebrafish embryos showed a remarkable heterogeneity and cancer cells presented higher dissemination patterns. **(A–C)** Confocal reconstructions of zebrafish embryos at 1 dpi and spatial dispersion pattern of xenografted human cells. **(A)** Non-leukemic cells. Yellow arrowheads correspond to fluorescent foci as a guidance to recognize them in leukemic xenografted embryos. Red asterisk represents the site of injection. **(B)** LPZ6-M4 **(C)** LPZ21 (B-ALL) sample. Cells were labeled with CFSE and engrafted in the pericardial space of zebrafish embryos at 48 hpf. A fluorescent signal, dorsal to the yolk, was detected at the digestive system in all xenografted embryos due to the metabolism of CFSE (*n* = 6 embryos per cell sample).

**Figure 3:**
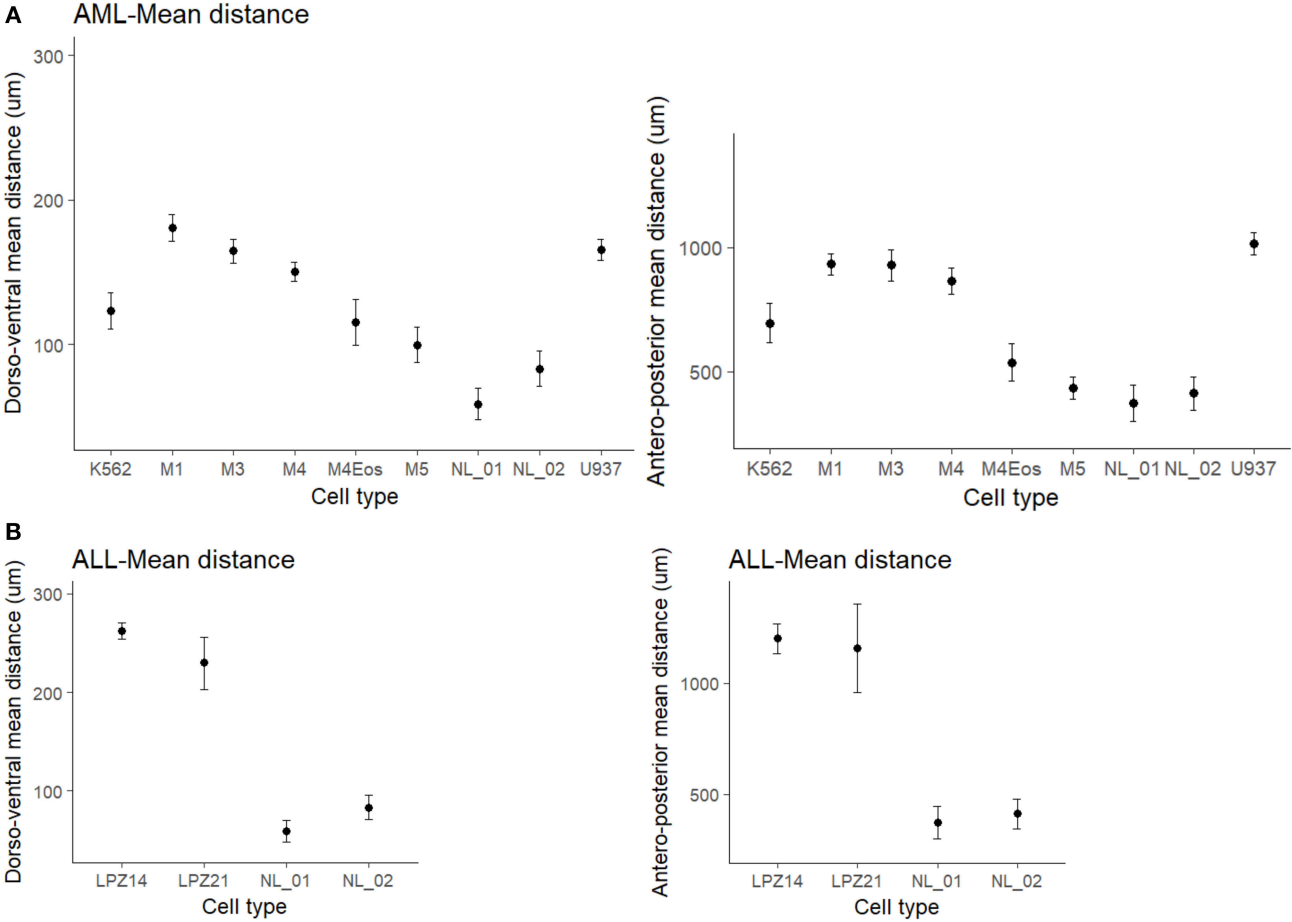
Mean migration distances of xenografted cells into zebrafish embryos at 1 dpi. **(A)** Differences of mean migration between myeloid leukemic and non-leukemic human cells toward the antero-posterior and dorso-ventral axis. **(B)** Differences of mean migration between lymphoid leukemic and non-leukemic human cells. Non-overlapping confidence intervals mean statistical significance. The comparison was established according to morphological and immunophenotypic similarities between samples from the same subtype based on FAB scheme. Migration differences were established based on a standard-deviational ellipse with 9999 permutations. NL, Non-leukemic cells.

**Figure 4:**
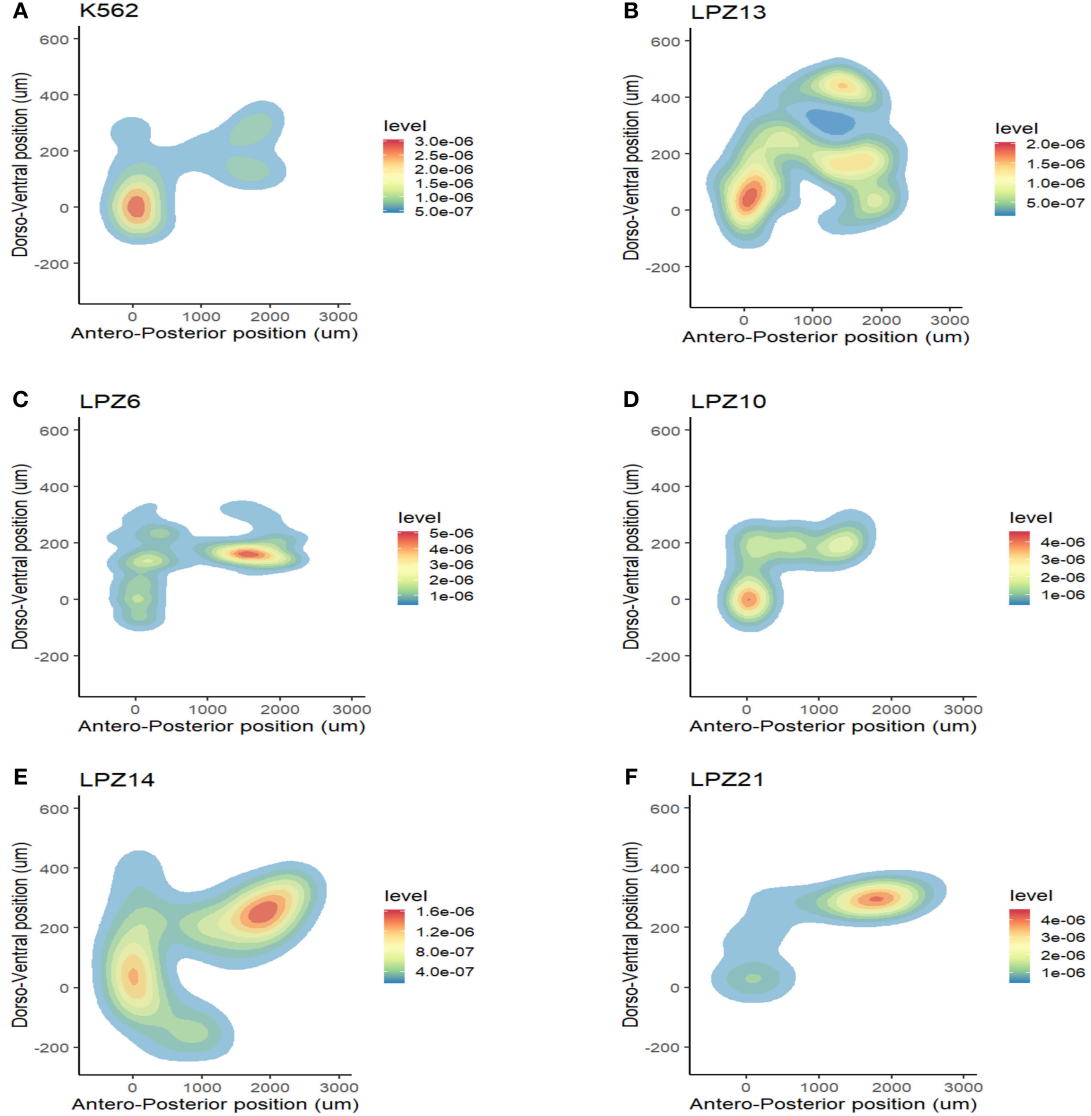
Inter-tumor heterogeneity in migration patterns. Spatial dispersion pattern of cell line xenografts did not resemble the distribution of primary leukemic cells in a complex microenvironment. **(A)** K562 cell line. K562 is a chronic myelogenous cell line in a blastic crisis. This cell line can be compared to the less differentiated myeloid leukemia subtypes (M0 and M1). **(B)** M1 sample. Cell line behavior does not resembles PDX behavior. **(C–F)** Spatial dispersion patterns between primary samples of the same leukemia subtype presented significant differences in their migratory potential. **(C,D)** Evidence of inter-tumor heterogeneity between AML-M4 samples. **(C)** LPZ6 **(D)** LPZ4. **(E,F)** Evidence of inter-tumor heterogeneity between B-ALL samples. **(E)** LPZ14 **(F)** LPZ21.

First, non-leukemic cells have the most restricted and narrowest migration (Figure 2A). In contrast, all the leukemic cells presented broader dispersion and migrated farther away from PCS (Figures 2B,C, 3). According to this analysis, less differentiated leukemia subtypes (LPZ13-M1, LPZ12-M3) have a greater mean migration distance in both, anteroposterior and dorsoventral axes (Figure 3A). Additionally, when comparing primary cells and cell lines from the same leukemia subtype we described multiple discrepancies in their behavior. Cell lines' capacity to migrate outside of PCS did not resemble the behavior of their PDX counterparts and they presented differences in their dissemination pattern (Figures 3A, 4A,B). Furthermore, PDX from the same leukemia subtype, such as M4 leukemias, presented heterogenic dispersion behaviors, and their dispersion potential is significantly different (Figures 3A, 4C,D). This highlight the importance of inter-tumor heterogeneity in order to understand acute leukemia biology.

On the other hand, in B-ALL, LPZ14 cells disseminated preferentially toward the rostral part of the embryo (Figure 4E). In contrast, LPZ21 exhibited one migration hotspot in the posterior region of the embryo and a narrower migration (Figure 4F). However, none of these differences were statistically significant (Figure 3B).

### Human Leukemic Cells Presented a Tropism Toward the Hematopoietic Tissue

**Figure 5:**
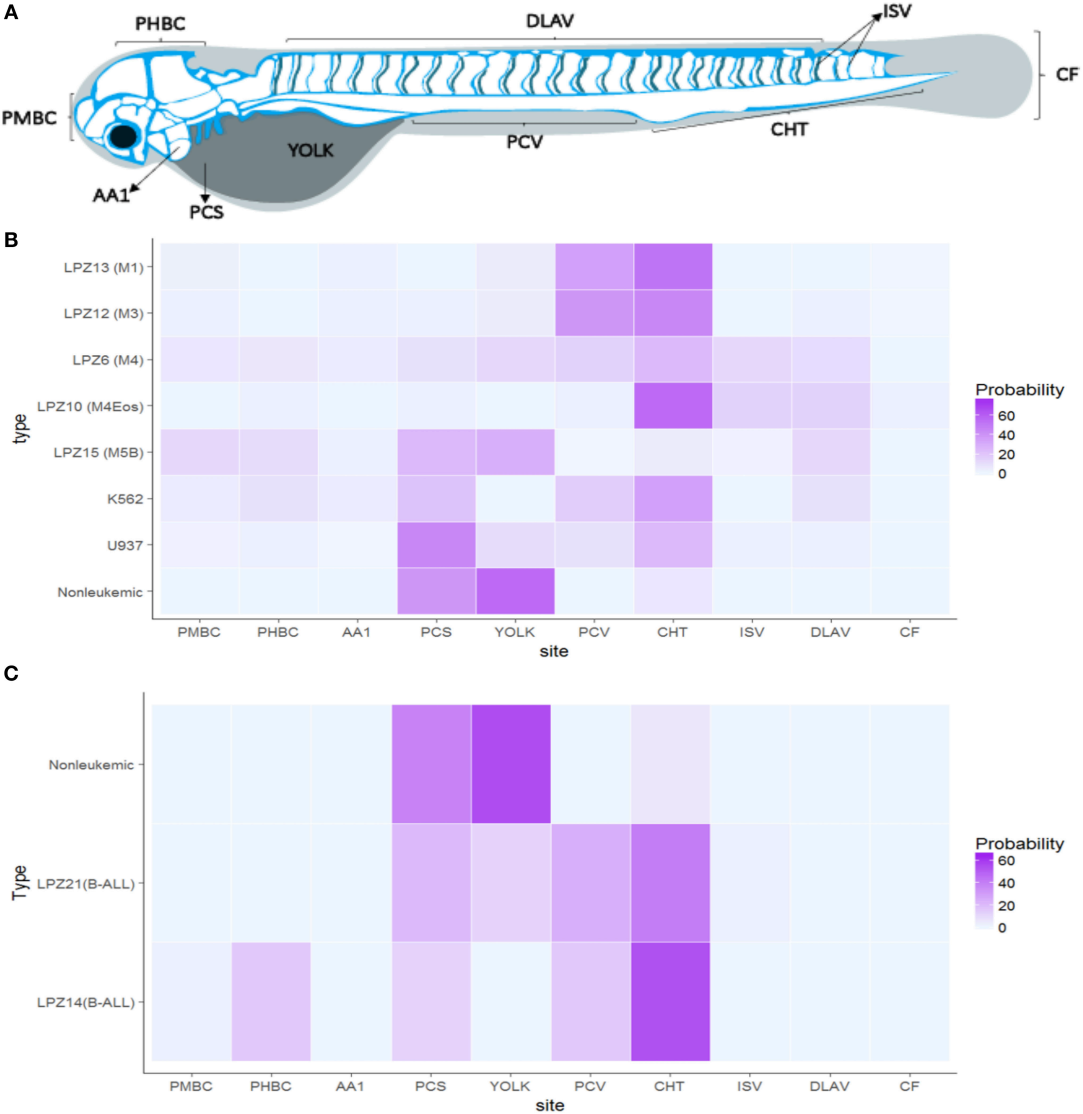
Acute leukemia xengorafts tropism. Human cells exhibited a clear tropism toward the hematopoietic tissue of zebrafish embryos. **(A)** Anatomic migration regions based on zebrafish vasculature and hemodynamics. **(B)** Probability (%) heat map of AML xenografts. **(C)** Probability (%) heat map of ALL xenografts. Significant differences were determined based on Bayesian 95% Highest Density Interval. Pericardial space (PCS), Yolk, Caudal Hematopoietic Tissue (CHT), Caudal Fin (CF), Intersegmental veins (ISV), Primordial Hindbrain Channels (PHBC), Posterior Caudal Vein (PCV), Primordial Midbrain Channel (PMBC), and Dorsal Longitudinal Anastomotic Vessel (DLAV) (*n* = 24 embryos per cell sample).

In order to determine if PDX exhibited a preferential site for migration, we calculated the probability of observing foci in 10 anatomical regions selected according to the zebrafish hemodynamics (Figure 5A). Non-leukemic cells were concentrated in the regions adjacent to the PCS (50%) suggesting invasiveness rather than metastasis (44). However, around 20% of the cells were able to travel far distances achieving the Caudal Hematopoietic Tissue (CHT) of the embryos, a hematopoietic site of zebrafish during the larvae stage.

In contrast, leukemic cells were able to migrate and establish in more regions. Less differentiated leukemia subtypes, LPZ13-M1 and LPZ-12-M3, presented the lowest probability of staying at PCS (10%), suggesting a higher intrinsic potential of these subtypes to intravasate and migrate. Both subtypes migrated preferentially to the CHT and the Posterior Cardinal Vein (PCV) showing a probability of 40% to be found in those regions. Similarly, K562 had a preferential migration toward the CHT (35%); however, the cells were more evenly distributed along the embryo, and a higher number of cells were found in the PCS compared to LPZ13-M1 and LPZ12-M3. Thus, K562 xenografts have a limited potential to migrate outside the site of injection. Similarly, more differentiated leukemia samples (LPZ6-M4, and LPZ15-M5) presented a higher number of foci at PCS and a more random distribution. However, LPZ10-M4Eos presented a clear tropism toward the CHT despite it is classified as a differentiated acute leukemia (Figure 5B). This suggests the presence of stem-like clones, which agrees with the high proportion of blasts found in the sample at the moment of the diagnosis (Table 1).

B-ALL samples presented considerable differences compared to non-leukemic cells. Both xenografts effectively intravasated and reached multiple medial and posterior regions of the embryos such as PCV and CHT, being the latter the most common place to find these cells (Figure 5C). However, LPZ14 cells were frequently found in the Primordial Hind Brain Channel (PHBC) and Primordial Mid Brain Channel (PMBC), contrary to the LPZ21 which was restricted to the medial and posterior region with a lower probability to establish in the most anterior portion. In addition, LPZ21 displayed more invasiveness events to the yolk and a greater proportion of human cells remain at PCS (32%). When comparing with non-leukemic cells, LPZ14-injected embryos presented a significantly higher probability in the PMBC and a significantly lower proportion of cells in the PCS. On the other hand, LPZ21 only presented a significantly greater migration toward PVC compared to control (Figure 5C).

Moreover, these results suggested a possible relationship between metastatic potential and patients' clinical outcome. Two (LPZ6-M4 and LPZ15-M5) of three patients with unsuccessful morphological remission presented a random distribution along the embryos, except LPZ13-M1. LPZ6-M4 and LPZ15-M5 had an abnormal karyotype while LPZ13-M1 presented a normal karyotype. Meanwhile, embryos with PDXs from patients with successful remission presented a clumped distribution toward de CHT. In the same way, B-ALL PDX with a worse prognosis and outcome (LPZ21) presented a random distribution, while LPZ14, with favorable outcome, showed a clear preference toward CHT. However, this is not conclusive due to the limited number of samples (Chi-sq = 0.3865).

In summary, our results demonstrated that malignant human hematopoietic and leukemic cells have a tropism toward CHT. In addition, we evidenced a heterogeneous cellular composition in each sample. This was proved by the capacity of a limited set of cells to leave the site of injection and invade immediate and distant regions. Also, PDX from patients with a worse prognosis and unsuccessful remission have a tendency to disseminate randomly along the fish.

### Clinical Prognostic Factors Do Not Correlate With Clinical Outcome

WHO classification scheme had replaced the FAB system and the latter has been considered obsolete for prognosis (9). Contrary to this proposition, our *in vivo* results showed that this system seems to have a clinical relevance, specifically to understand leukemia's behavior in a complex microenvironment. FAB system is based on cell morphology and blast counts without considering other clinical variables to evaluate the prognostic significance (45). For that reason, we determined the relation between other hematological parameters and the clinical outcome of the patients. According to our results, there was not a significant correlation between the current prognostic factors and the morphological remission after the induction treatment (*p*-value >0.05 for all prognostic factors).

### Patients With Numerous LSC Presented Poor Clinical Outcomes

**Figure 6:**
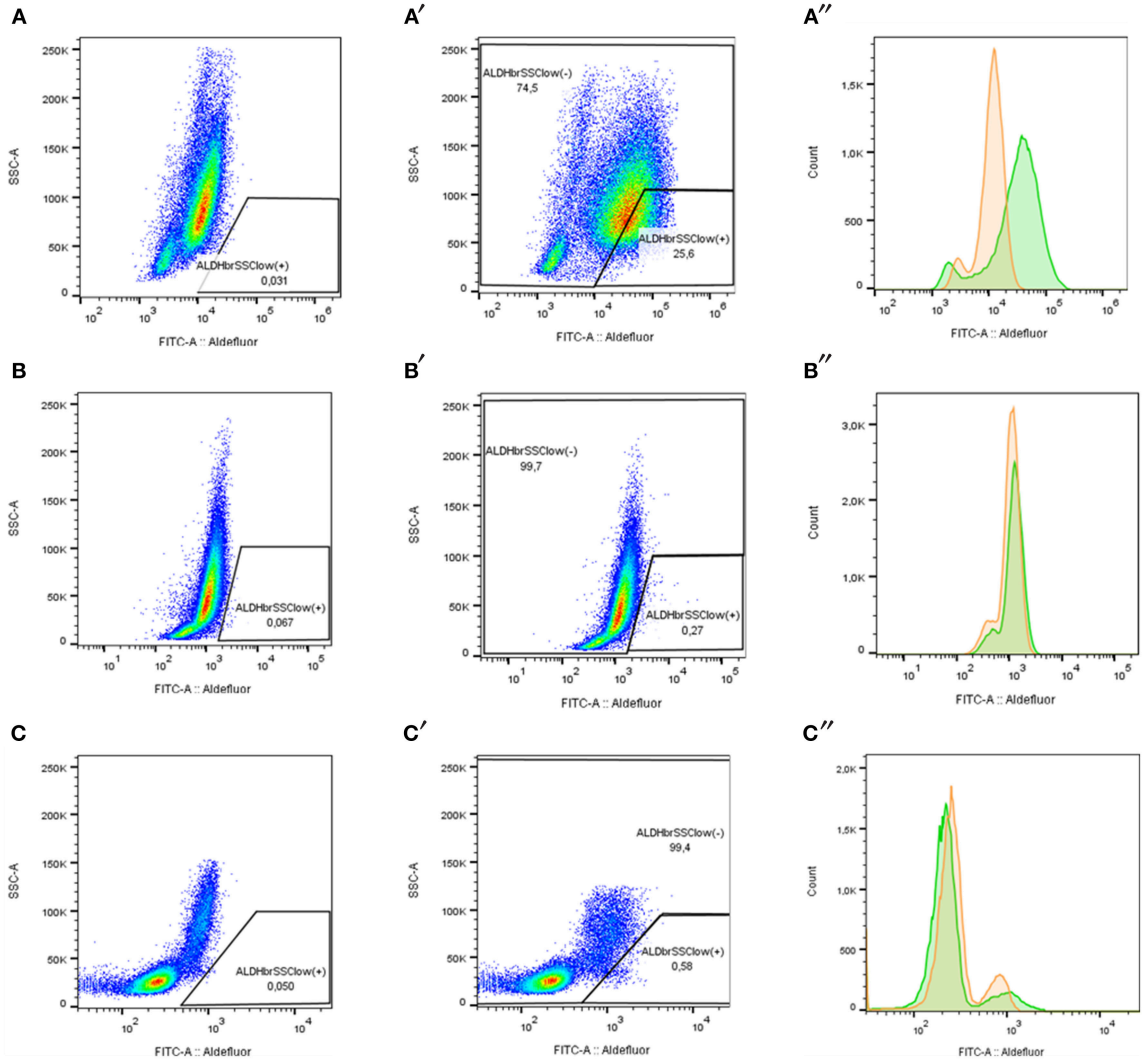
ALDH pattern determination according to ALDH expression in acute leukemia samples stained with ALDEFLUOR reagent. **(A–C)** ALDH^bright^SSC^low^ (+) cells percentage with Aldefluor inhibitor (DEAB+). **(A′-C″)**. ALDH^bright^SSC^low^ (+) cells and ALDH^bright^SSC^low^ (-) cell percentage without inhibitor (DEAB-). **(A″-C″)** FITC fluorescence distribution determines ALDH activity in human samples. DEAB+ (orange) and DEAB- (green). **(A–A″)** LPZ6 (ALDH-numerous). **(B–B″)** LPZ12 (ALDH-rare). **(C–C″)** Non-leukemic cells from a healthy donor (negative control).

Another indicator of patients prognosis and clinical outcome is tumor heterogeneity (46). For that reason, we delimited the proportion of potential LSC candidates using ALDH activity and CD34 expression as discriminatory parameters. LSC population was defined as ALDH^bright^SSC^low^CD34^+^ and each sample was classified as ALDH-numerous pattern [>1.9% ALDH^bright^SSC^low^ cells] or ALDH-rare pattern [<1.9% ALDH^bright^SSC^low^ cells] as previously described (18, 19) (Figure 6). This evaluation was performed in human primary leukemic and non-leukemic cells (except in sample LPZ10) and in cell lines.

According to our results, ALDH^bright^SSC^low^CD34^+^ cells corresponded to a small proportion of the whole tumor burden, but this fraction varied according to the leukemia subtype. In AML, less differentiated sample, LPZ13-M1, showed the highest proportion of LSC with 17.28%. In contrast, its cell line counterpart, K562 presented one of the lowest amounts of LSC (0.06%). Similarly, when comparing both promonocytic leukemia, LPZ15-M5 and U937, the number of cells expressing both markers was greater in primary cells than in the cell line, with 0.47%, and 0.25%, respectively. The remaining AML primary samples also presented dissimilar proportions of ALDH^bright^SSC^low^CD34^+^ population and seemed to be consistent with risk stratification. Patient with an adverse prognosis (LPZ6-M4) presented the second highest percentage of LSC in AML with 13.08%, and the patient with a favorable prognosis (LPZ12-M3) presented the lowest proportion with a 0.03% (Table 3). Similarly, in B-ALL, adverse sample, LPZ21, had a higher proportion of LSC (23.78%) compared to a standard risk sample LPZ14 with 1.18% (Table 3). This highlights that primary cells are strikingly heterogeneous and reveals that cell lines are far from mirroring primary leukemic cell features.

**Table 3:** LSC proportion in leukemic samples based on ALDH activity.

**Patient**	**ADLH pattern**	**ALDH bright SSClow (%)**	**CD34+(%) in ALDH bright SSClow population**	**ALDH bright SSClow CD34+ (%)**
**AML**				
LPZ6	Numerous	25.6	51.1	13.08
LPZ12	Rare	0.27	11.3	0.03
LPZ13	Numerous	37.9	45.6	17.28
LPZ15	Rare	1.58	29.7	0.47
**ALL**				
LPZ14	Rare	1.74	68	1.18
LPZ21	Numerous	24.96	24.83	23.78
**CELL LINE**				
K562	Numerous	31.3	0.19	0.06
U937	Rare	0.96	26.4	0.25

On the other hand, ALDH activity is a potential predictive factor, due to its association with chemotherapy resistance and patient relapse (47, 48). According to our results, none of the patients with numerous LSC (>1.9% of tumor bulk) achieved complete remission during induction treatment, while two of three patients with rare pattern (<1.9% of tumor bulk) did these observations agrees with the dissemination potential of the cancer cells into the zebrafish embryos. In addition, patients with ALDH-numerous samples showed higher bone marrow blast infiltration and correlates in most of the cases with the tropism of hematopoietic niche observed in the fish. However, a slight correlation was found between unsuccessful morphological remission and LSC subpopulation (*R*^2^ = 0.625 *p*-value = 0.05414, *n* = 6).

### An Integrative Leukemic Model Predicts Clinical Outcome

**Figure 7:**
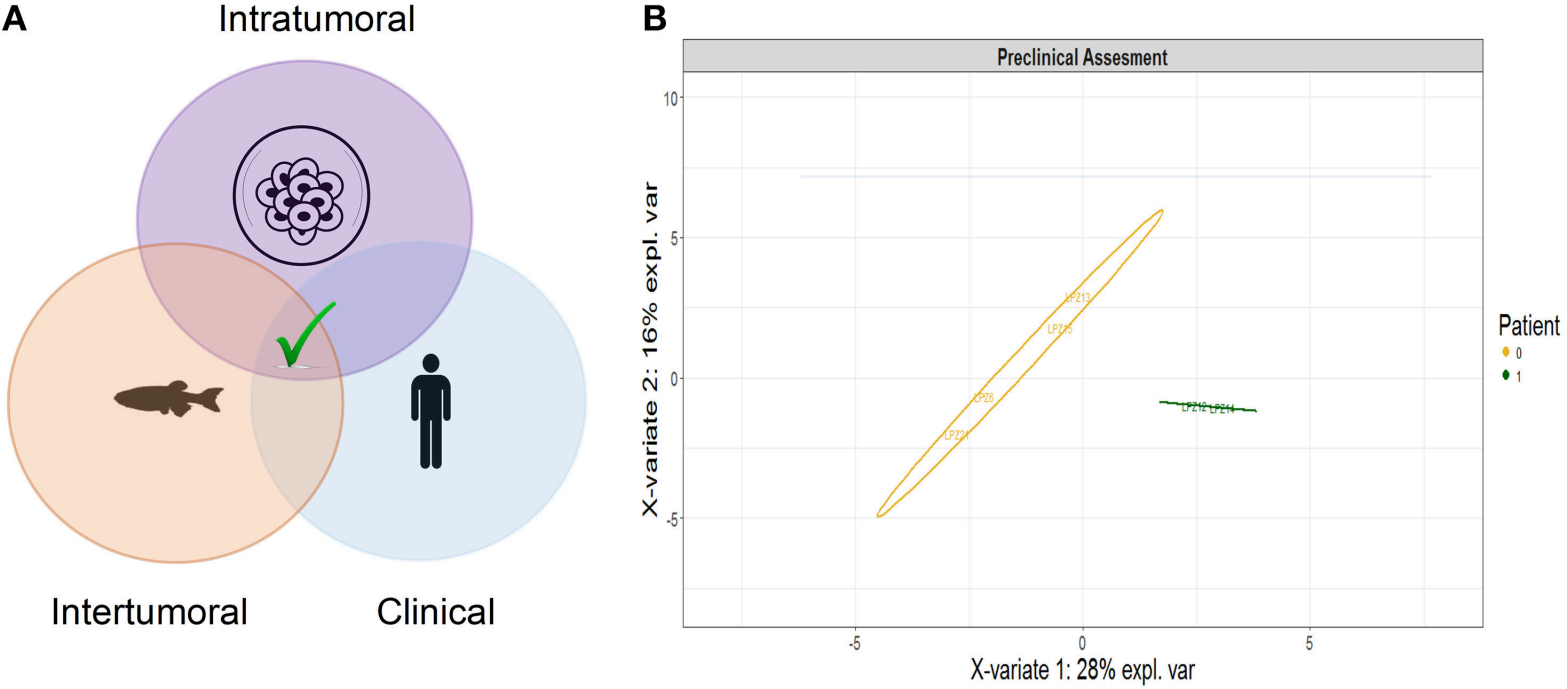
PLS-DA score plot of the clinical outcome predicted by the integrative model. **(A)** Infographic scheme of the mathematical model **(B)** Clinical information, LSC characterization and *in vivo* behavioral analysis explains 44% of the variance between patients newly diagnosed with acute leukemia and discriminate accurately the patients into a successful and unsuccessful clinical outcome after induction treatment. Component 1 discriminated better the patients according to their clinical outcome into successful or unsuccessful remission. The most influential variables with respect to the clinical outcome corresponded to ALDH activity, LSC proportion, and migration along zebrafish embryos. 1, successful morphological remission; 0, Unsuccessful morphological remission.

Developing new and better tools for the stratification of patients with acute leukemia has become a clinical priority during the last decades. In this study, an accurate predictive model was established consolidating clinical features, LSC characterization and *in vivo* behavioral analysis to predict patients' remission outcome and improve risk assignment (Figure 7A). We performed a partial least- squares-discriminant analysis (PLS-DA) utilizing the mixOmics package in R. This pilot study established a new tool that discriminates accurately the patients according to their clinical outcome based on the integration of different aspects of the disease (Figure 7B). The relationship between the successive pairs of scores is robust and explains 48% of the variation of the response. A higher proportion of LSC candidates was the best indicator of unsuccessful remission, while higher migration to Primordial Hind Brain Channel (PHBC) region and Mandibular Arch (AA1) corresponded to the best predictive factors of a favorable remission. On the contrary, traditional clinical variables currently used for risk stratification had the lowest weights in our study, suggesting that these variables might be insufficient for clinical outcome prediction if are analyzed independently from tumor composition and leukemic cell behavior.

## Discussion

Currently, acute leukemia risk stratification is based on morphological features, cell markers, and cytogenetic aberrations. However, in adults, most of the clinical features gathered regularly have a weak association with the clinical outcome, especially in patients classified in an intermediate risk group (6, 9, 10). For that reason, the importance of having additional prognostic markers and to understand leukemia etiology and biology, especially for intermediate risk patients is imminent. In our study, and to our knowledge, for the first time, a completely different approach integrating clinical information, tumor cell characterization and *in vivo* PDX behavioral analysis has been proposed as a reliable tool to understand acute leukemia and to predict clinical response accurately.

**Figure 8:**
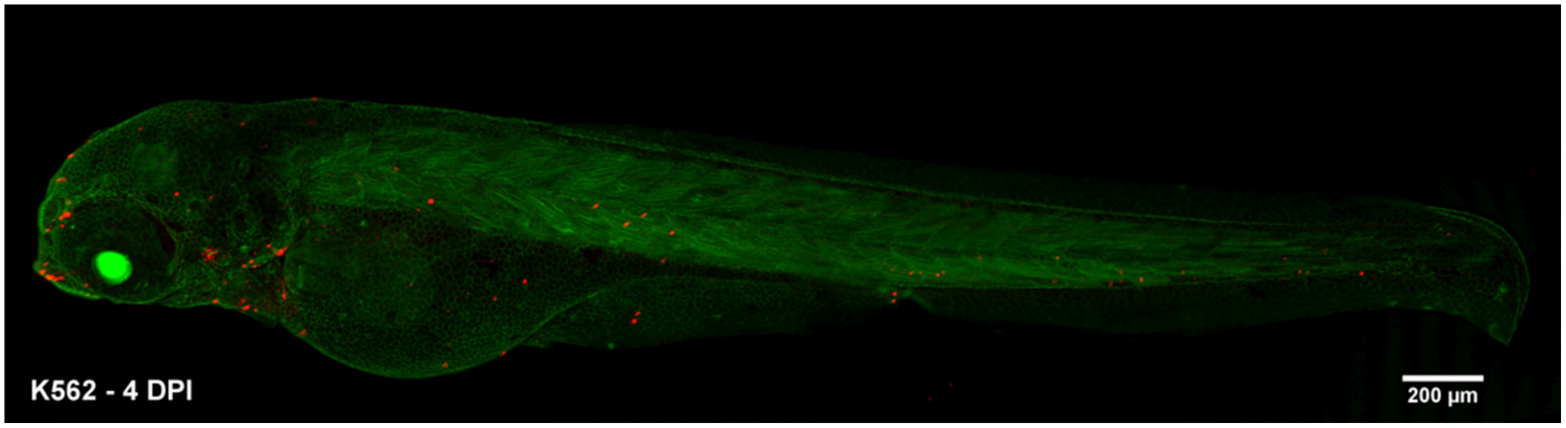
Alternative xenograft assay for long-term analysis. Transgenic β-actin zebrafish embryos were injected with k562 cells stained with C-DiL at 48 hpf. The visualization of the cells was performed at 4 dpi. At this time the cells were easily detected and maintained their tropism toward the hematopoietic tissue of the fish. K562 xenografted cells at 4 dpi.

First, we evaluated the behavior of human acute leukemias in a complex microenvironment. In this study, we demonstrated that PDX into zebrafish embryos is a suitable model to study leukemia biology. Zebrafish allowed the tracing of human leukemic cells in a short period (1 to 2 dpi) using CFSE. At this time, cell survival, early (intravasation) and late (extravasation and colonization) steps of metastasis and cellular tropism were assayed as demonstrated before with other types of cancers like breast cancer, colorectal cancer, and fibrosarcoma (24, 49). Despite, our methodology was limited to evaluate the behavior of human leukemic cells during longer periods, further analysis up to 4–7 dpi are also easily performed using similar methodologies as xenografting C-DiL stained cells into transgenic β-actin embryos (Figure 8) or as done in previous studies (26, 50, 51). As a complete organism zebrafish has a number of important advantages over the traditional murine model and has become an alternative to overcome mice model limitations. These advantages have been reviewed recently (29, 52).

In particular, for the study of hematopoiesis and hematological diseases the zebrafish model is considered a suitable tool due to its capacity to reproduce many of the tissues and molecular interactions with leukemic cells, resulting in a reliable reproduction of the human disease (22, 29, 49). Also, many molecular and cellular components involved in tumorigenesis are highly conserved between zebrafish and human, making the model clinically relevant (53).

At the level of genome sequence conservation, 82% of human disease genes have at least one zebrafish ortholog (54).In addition, the fish have all major hematopoietic cell lineages, which are generated by similar developmental pathways. There is also remarkable conservation of transcription factors, signaling pathways, and functional proteins involved in hematopoiesis and leukemogenesis (29, 55–57).

The fish also provides a novel way of studying human tumors *in vivo* allowing real-time intravital imaging of injected human leukemic cells and allows direct observation of cancer formation and progression in the living animal. At the same time, typical behaviors associated with the disease such as tumor angiogenesis, leukocyte recruitment, and inflammation are easily assayed (26, 58, 59).

In our study, we were able to demonstrate that the zebrafish provides human cells with an appropriate niche for them to survive. We and others have proved that not only cancer cell lines can be detected into embryos, but also primary human cancer and healthy cells exhibited the ability to establish and migrate (24, 60–62). Moreover, other investigations have demonstrated that zebrafish maintain the cancerous phenotype of leukemic cell lines (63, 64). In this study, xenografted cancer cells had higher survival rates, reached farther distances and colonized multiple regions in the zebrafish, which agrees with previous research. This represented a better adaptation of leukemic cells to different microenvironmental conditions, which can be associated with cancerous features (65). Furthermore, this suggested that this system maintains the original phenotype of the tumor preserving the original features and evidence that cancer cells are less dependent on niches' characteristics probably by their capacity to develop some mechanisms that allow them to customize the niche to ensure their survival (66).

On the other hand, multiple investigations have demonstrated that animal models mimicked the disease (21). Zebrafish cancer models of myeloma, breast cancer, rhabdomyosarcoma, and endocrine tumors present molecular and histopathological conservation with their human cancer counterparts (23, 49, 67, 68). For example, Mercatali et al. demonstrate that primary cells of breast cancer in the zebrafish embryos showed a behavior resembling that of the patient's medical history (49). While Tang et al. observed that the evolution of metastatic potential of melanoma in zebrafish is consistent with that reported for human melanoma (69).In addition, some others have shown that human tumor cells are capable to proliferate and interact with vascular tissues in zebrafish embryos (53, 58). Recently, some researchers showed that zebrafish is a high sensitivity model for chemotherapy screening and demonstrated the capability of PDX in the fish to anticipate relapse within 3 m to 6 m after surgery in adenocarcinoma (70).

In our study, the dissemination pattern and metastatic potential of leukemic cells were well characterized. Previous researches with murine models revealed that leukemic cells repopulated the bone marrow at greater frequency followed by spleen and peripheral blood (7). Our results demonstrated that engrafted cells in zebrafish embryos intravasated and circulated within the embryonic vasculature to finally establish preferentially in the CHT, which recapitulates a bone marrow-like metastatic niche. This points out the importance of the hematopoietic niche as a supportive tumor microenvironment. In addition, xenografted cells homed first to CHT, the site of leukemogenesis, before progressing to other anatomical regions.

The preferential establishment toward CHT can be explained by the fact that this tissue has stromal features, where hematopoietic cells expand and a perivascular niche, where stem cells are anchored and regulated (71, 72). In addition, CHT sends homing cues, such as chemokines CXCL12a (stromal cell-derived factor 1) and CXCL8 (MCP2) and other molecules involved in cell development, for example, Erythropoietin, Thrombopoietin, and Notch signaling (39, 67, 73). CHT also produces other supportive cytokines associated with hematopoiesis, leukocyte trafficking, inflammation, and neutrophil retention (39, 74). Some studies had demonstrated that transplanted human cancer cells are capable to respond to zebrafish cues, modulate the niche, and take advantage of the zebrafish stroma during cancer progression (53, 75). Thus, once more the zebrafish seems to be a reliable system to help to dissect the mechanisms underlying dissemination and metastasis in leukemia and other different types of cancer.

On the other hand, one essential challenge in understanding acute leukemia is the inherent heterogeneity of tumor cells subpopulations within each patient. Cancer tumors are characterized by a wide diversity of cell types that are differentiated by developmental stages, genetic mutations and altered cell programs that result in functional diversity of cell subpopulations. The behavior within the fish presented diverse manifestations and striking heterogeneity among primary human cells. For that reason, our study also incorporates tumor heterogeneity in order to better understand aggressiveness and invasiveness phenotypes. For intra-tumor heterogeneity, LSC paradigm suggests that the tumor bulk corresponds to cells with a high proliferative potential not involved in leukemogenesis. However, a small portion of tumor cells has stem cell characteristics according to our LSC characterization. These cells express genes that regulate quiescence and self-renewal, which are associated with carcinogenic and metastatic events (76, 77). Our results confirm this asseveration. Injected samples exhibited heterogenic cell subpopulations in which some human cells remain at the site of injection, while some others were able to migrate and settle in distant regions. This evidenced the use of zebrafish embryos for the study of clonal evolution which is another striking aspect involved not only in tumor initiation but also plays an important role in intratumor heterogeneity. Also, this opens novel opportunities for further studies in order to quantify the cancer heterogeneity and determine the characteristics of each cell subpopulation *in vivo* to understand metastatic mechanisms that can be used for the development of new treatments.

On the other hand, inter-tumor heterogeneity was assayed based on risk group and FAB classification (immunophenotype). Despite the limited number of samples, PDX from AML-M4 and ALL-B were used for direct comparisons between same leukemia subtypes. Our results demonstrated that the dissemination pattern resembled differences among patients. This agrees with the differential behaviors between leukemic cells with distinct immunophenotypes shown in mice (76). In addition, zebrafish model allows the evaluation of differential behaviors among different leukemia subtypes which in most cases resembles the aggressiveness of human leukemia and agrees with current classification systems. This provides a platform for the development of more selective and appropriate personalized therapy.

Additionally, our data enable to associate the behavior of PDX along with the clinical outcome of the patients, where a random dissemination trend to have a worse response after induction chemotherapy. Moreover, human cancer cells from samples with a random migration seem to have an enhanced capability to spread through different regions along the zebrafish. However, future trials evaluating cancer heterogeneity should establish a clear relationship between the dissemination pattern within the fish and the clinical outcome for each leukemia subtype.

In agreement with other studies (12, 24), we demonstrated that the behavior of primary human cancer cells in zebrafish embryos could be used to predict metastasis, aggressiveness, and prognosis. Each type of cell revealed particular features to survive and settle in different microenvironments, associated with their differentiation potential. These results suggested a disparity between aggressiveness potential of PDX related to differentiation and different metastatic potential within tumor cell populations. We ratify that PDX is as a new promising tool to mirror tumor heterogeneity and cancer microenvironment of the primary tumor. In addition, this highlights the importance of implementation of *in vivo* models and the applicability of PDX alternative assessments since cell lines have different behavior and did not resemble the biology of primary cells.

Regarding leukemia biology, our results show that xenografts from less differentiated samples and samples with adverse cytogenetic aberrations exhibited higher LSC, a greater survival, migration and metastatic potential at 1 dpi. In this context, aggressiveness in our behavioral analysis agrees with both FAB and WHO classification; thus, current classification systems together have a clinical significance, but independently they remain insufficient. Although we had a limited number of samples, our results suggested that the existing prognostic factors by themselves, analyzed in this study, might not have strong predictive potential. This tendency agrees with other observations from different studies in regard to the predictive power of these clinical features. Thus, despite recent advances, clinical outcome prediction remains misleading (78).

On the other hand, an *ex vivo* heterogeneity approach was assessed. We demonstrated that the samples with the highest ALDH activity contained an enriched CD34^+^ population, suggesting higher stemness and immaturity in the tumor burden. Our observations can be associated with the migration pattern into the zebrafish embryos and a poor prognosis and unfavorable clinical outcome (48). Although we observed that ALDH characterization by itself is insufficient to accurately predict the response of the patients, we were able to establish that tumor characterization agrees with the behavior of human cancer cells in a complex microenvironment. These findings are consistent with LSC paradigm, which consider that a set of tumor cells with “stem cell-like” features are able to maintain all the neoplastic populations and would be responsible for relapse and chemotherapy resistance (17, 79). In this context, higher frequency of LSC is a good indicator of failure in remission and, high ALDH activity cells have been related to high rates of metastasis and tumor growth as previously demonstrated (19, 48). Furthermore, each ALDH pattern suggested different prognostic values in clinical contexts. According to our results, there is a tendency for numerous patterns to have random migration into the fish and a worse clinical outcome after induction chemotherapy compared to a rare pattern, and this is directly implicated in patients' prognosis. Hoang et al. stated that numerous ALDH pattern is related to reduce disease-free and overall survival rates in contrast to the rare pattern, which supports our results (18).

In summary, despite karyotype being one of the most important prognostic factors in *de novo* leukemia (80), additional tests are needed to provide a full picture of the behavior of the leukemic cells and its relation to the prognosis. Understanding the cellular behavior that drives leukemia progression is highly relevant to diagnosis, risk stratification and the development of new therapies. For that reason, the *ex vivo* and *in vivo* approaches together performed in this study showed an important relation between the aggressive behavior of leukemic cells and the outcome of the patients. We proved that the behavior of PDX within the fish depended mainly on their intrinsic features, but also seemed to have a closer relationship with the fish microenvironment. Additionally, we demonstrated that the study of early and late steps of metastasis in zebrafish is a rapid and robust approach that allowed the visualization of this process in a short period of time. Also, the model had multiple advantages for the use of primary samples which helps with a reliable understanding of the disease. It is a valuable tool to study leukocytes biology and to model human leukemia, due to similar molecular mechanisms and a high degree of conservation between species. However, in order to have an accurate prediction of the clinical outcome of patients was necessary to integrate clinical, *in vivo* behavioral and *ex vivo* cellular features of acute leukemia. Taken together, all the variables of our multifactorial evaluation highlight the importance to understand the disease from different approaches in order to achieve an accurate quantitative prediction of the prognosis and an objective stratification of risk groups. Despite our sample number was reduced the approach seems to be robust enough. This study is the first glimpse for the implementation of this model in the definition of the clinical prognosis and outcome of patients with acute leukemia. However, further analysis are required for its clinical application

Finally, our improved model is robust, easy, and reliable for pre-clinical acute leukemia studies and provides novel insights in understanding the relevant predictive factors of clinical outcome. In addition, this study opens a wide opportunity for further studies to decipher the role of tumor heterogeneity, malignant cellular mechanisms, and leukemic microenvironment interaction in the prognosis of adult patients.

## Author Contributions

ZG-A and VA: study conception and design. AN-J, NB, HI, and MG-G: acquisition of data. LA and GQ: sample collection and clinical information attainment. NB and JG: flow cytometry supervision and material supply. AN-J and MG-G: analysis and interpretation of data. AN-J, MG-G, and ZG-A: drafting of manuscript. ZG-A, GQ, JG, and VA: critical revision.

### Conflict of Interest Statement

The authors declare that the research was conducted in the absence of any commercial or financial relationships that could be construed as a potential conflict of interest.
